# Mesoporous Bioactive Glass-Incorporated Injectable Strontium-Containing Calcium Phosphate Cement Enhanced Osteoconductivity in a Critical-Sized Metaphyseal Defect in Osteoporotic Rats

**DOI:** 10.3390/bioengineering10101203

**Published:** 2023-10-16

**Authors:** Seemun Ray, Ulrich Thormann, Inga Kramer, Ursula Sommer, Matthäus Budak, Matthias Schumacher, Anne Bernhardt, Anja Lode, Christine Kern, Marcus Rohnke, Christian Heiss, Katrin S. Lips, Michael Gelinsky, Volker Alt

**Affiliations:** 1 Laboratory of Experimental Trauma Surgery, Justus Liebig University, 35390 Giessen, Germany; seemun.ray7@gmail.com (S.R.); ulrich.thormann@chiru.med.uni-giessen.de (U.T.); inga.kramer@gmx.de (I.K.); ursula.sommer@chiru.med.uni-giessen.de (U.S.); matthaeus.budak@chiru.med.uni-giessen.de (M.B.); christian.heiss@chiru.med.uni-giessen.de (C.H.); katrin.s.lips@chiru.med.uni-giessen.de (K.S.L.); 2 Department of Trauma Surgery, University Hospital Giessen-Marburg GmbH, Campus Giessen, 35390 Giessen, Germany; 3 Centre for Translational Bone, Joint, and Soft Tissue Research, Faculty of Medicine, University Hospital Carl Gustav Carus, Technische Universität Dresden, 01307 Dresden, Germany; schumacher.m@posteo.de (M.S.); anne.bernhardt@tu-dresden.de (A.B.); anja.lode@tu-dresden.de (A.L.); michael.gelinsky@tu-dresden.de (M.G.); 4 Institute of Physical Chemistry, Justus Liebig University Giessen, 35392 Giessen, Germany; christine.kern@phys.chemie.uni-giessen.de (C.K.); marcus.rohnke@phys.chemie.uni-giessen.de (M.R.); 5 Department of Trauma Surgery, University Hospital Regensburg, 93053 Regensburg, Germany

**Keywords:** fracture defect, biomaterial, mesoporous bioactive glass, strontium, osteoporosis

## Abstract

In this study, the in vitro and in vivo bone formation behavior of mesoporous bioactive glass (MBG) particles incorporated in a pasty strontium-containing calcium phosphate bone cement (pS100G10) was studied in a metaphyseal fracture-defect model in ovariectomized rats and compared to a plain pasty strontium-containing calcium phosphate bone cement (pS100) and control (empty defect) group, respectively. In vitro testing showed good cytocompatibility on human preosteoblasts and ongoing dissolution of the MBG component. Neither the released strontium nor the BMG particles from the pS100G10 had a negative influence on cell viability. Forty-five female Sprague–Dawley rats were randomly assigned to three different treatment groups: (1) pS100 (*n* = 15), (2) pS100G10 (*n* = 15), and (3) empty defect (*n* = 15). Twelve weeks after bilateral ovariectomy and multi-deficient diet, a 4 mm wedge-shaped fracture-defect was created at the metaphyseal area of the left femur in all animals. The originated fracture-defect was substituted with pS100 or pS100G10 or left empty. After six weeks, histomorphometrical analysis revealed a statistically significant higher bone volume/tissue volume ratio in the pS100G10 group compared to the pS100 (*p* = 0.03) and empty defect groups (*p* = 0.0001), indicating enhanced osteoconductivity with the incorporation of MBG. Immunohistochemistry revealed a significant decrease in the RANKL/OPG ratio for pS100 (*p* = 0.004) and pS100G10 (*p* = 0.003) compared to the empty defect group. pS100G10 showed a statistically higher expression of BMP-2. In addition, a statistically significant higher gene expression of alkaline phosphatase, osteoprotegerin, collagen1a1, collagen10a1 with a simultaneous decrease in RANKL, and carbonic anhydrase was seen in the pS100 and pS100G10 groups compared to the empty defect group. Mass spectrometric imaging by time-of-flight secondary ion mass spectrometry (ToF-SIMS) showed the release of Sr^2+^ ions from both pS100 and pS100G10, with a gradient into the interface region. ToF-SIMS imaging also revealed that resorption of the MBG particles allowed for new bone formation in cement pores. In summary, the current work shows better bone formation of the injectable pasty strontium-containing calcium phosphate bone cement with incorporated mesoporous bioactive glass compared to the bioactive-free bone cement and empty defects and can be considered for clinical application for osteopenic fracture defects in the future.

## 1. Introduction

The treatment of critical-sized bone defects is still a major challenge, especially in the context of systemically diseased bone tissue such as in case of osteoporosis. Injectable bone cements that are biodegradable and bioactive with appropriate mechanical strength have gained momentum in the treatment of bone defects after fractures in the last decades. Calcium phosphate bone cements (CPCs) are known for their excellent osteoconductivity, biocompatibility, and mechanical strength [1]. Since their discovery in the early 1980s, a variety of cement formulations have been described [2]. Recent developments include injectable cement formulations with extended shelf life [3,4] that are optimized for minimally invasive fracture treatment. Another recent strategy is the integration of bioinorganics into CPCs [5], in order to achieve a localized delivery of therapeutically active inorganic ions to trigger a biological effect in the defect region.

Osteoporosis is characterized by an impaired balance of bone resorption and bone formation by osteoclasts and osteoblasts, respectively. Since strontium ions (Sr^2+^) have been shown to act both as an inhibitor of bone resorption and as a stimulus of bone formation in vitro and in vivo [6,7,8], they have been proposed to counterbalance the impaired cellular processes and have gained considerable attention for the local treatment of osteoporotic bone defects. As such, strontium(II) has been included in CPC formulations applying different routes [9]. We previously reported the strontium(II) modification of a CPC by the complete substitution of CaCO_3_ by SrCO_3_ in the α-tricalcium phosphate-based cement precursor mixture of a hydroxyapatite-forming CPC (100% substitution: “S100”) [10]. As demonstrated, the CPC “S100” releases biologically effective doses of Sr^2+^ in vitro by the stimulation of human bone marrow-derived mesenchymal stromal cell proliferation and osteogenic differentiation as well as by the attenuation of osteoclastic resorption [11,12]. After implantation in a critical-sized metaphyseal bone defect model in overariectomized rats, mimicking osteoporotic bone fractures, an increased mass of new bone was observed for the CPC “S100” in comparison to the strontium(II)-free CPC. This was attributed to the release of Sr^2+^ from the “S100” in vivo, proven by time-of-flight secondary ion mass spectrometry (TOF-SIMS) analysis [13]. Beyond improvement of the biological properties, the strontium(II) modification resulted in a significant increase in the compressive strength and an increased radiopacity of the CPC [10]. The combination of the “S100” CPC precursor powder with a biocompatible oil-based carrier liquid resulted in a premixed, storable CPC paste (“pS100”) that is minimally invasive, which is applicable, for instance, in balloon kyphoplasty, while maintaining the advantageous properties introduced by the strontium(II) modification [14].

However, although they are bioresorbable by osteoclasts [15], the slow degradation rate of hydroxyapatite-forming CPCs in vivo remains a matter of concern. Also, the success of the CPC “S100” was limited, as 6 weeks after implantation in the rat osteoporotic defect model, no visible material degradation was observed [13]. A strategy to accelerate the degradation of such CPCs, to provide sufficient space for the influx of cells, is the introduction of other quickly degrading biomaterials that act as porogens introducing macroporosity. Polymeric microparticles that undergo hydrolytic degradation [16,17] or are water-soluble [18] have been investigated for that purpose. In this work, we followed a different strategy by combining the CPC with bioactive glass. Bioactive glasses are another promising group of bone repair materials, showing excellent bioactivity, biocompatibility, and tight binding to bone and soft tissue [19,20,21]. In contrast to CPC, bioactive glasses degrade much faster by dissolution. The ionic dissolution products, mainly calcium, silicon, and phosphate ions, have been shown to stimulate biological processes involved in bone regeneration [22]. In addition to conventional, melt-derived Bioglass, sol-gel synthesis of bioactive glasses has been developed and advanced to obtain materials with a highly ordered porosity on the mesoscale, resulting in a vastly increased surface area and further increased degradability. These are termed mesoporous bioactive glasses (MBGs) [23,24].

Recent studies have suggested that composite cements comprising MBG particulates embedded in a CPC may exhibit the formation of macropores within the cement matrix by gradual MBG degradation in such composites. This was indicated by an increasing porosity over time and the observation of irregular pits along the surface during in vitro incubation [25,26]. Thus, the aim of the present study was to investigate the degradation of CPC-MBG composites and its impact on bone regeneration in vivo. MBG particles were mixed into strontium(II)-modified, injectable CPC “pS100” with a ratio of 10 wt.%, and the resulting composite “pS10010G” was compared to the MBG-free “pS100”. After confirming cytocompatibility and the release of bioactive ions in vitro, both materials were evaluated in a critical-sized metaphyseal bone defect model in osteopenic rats. We tested the hypothesis that the integration of MBG particles in the “pS100” results in the formation of pores within the implanted material, which allows for tissue ingrowth and enhances the release of ions, with the potential to accelerate the osteogenic performance.

## 2. Materials and Methods

### 2.1. General Considerations

Animal procedures were performed in full compliance with the institutional and German protection laws and after approval by the local animal welfare committee (Ref. number: V54–19c 20/15–FU/1121). Forty-five female Sprague–Dawley rats were randomly assigned to three different procedure groups: (1) strontium-modified calcium phosphate paste cement with bioactive glass (pS100G10) (*n* = 15), (2) strontium-modified calcium phosphate paste cement (pS100) (*n* = 15), and (3) empty defect control (*n* = 15). An osteopenic bone status was induced by bilateral ovariectomy combined with a multi-deficient diet [27,28,29]. After three months, a 4 mm defect in the distal femur metaphysis was created, and the fracture was stabilized with a mini-plate [30]. The created fracture-defect was packed with either pS100G10 or pS100 or left empty. After six weeks, the animals were euthanized and the femurs removed, and histomorphometrical analysis including immunohistochemistry, enzyme histochemistry, molecular-biological analysis, and TOF-SIMS analysis for the detection of strontium, calcium, and collagen was performed. In the case of plate failure, e.g., breakage or loosening, specimens were not taken to further analysis.

### 2.2. Preparation of pS100 and pS100G10

The strontium (II)-modified calcium phosphate cement paste (pS100) was prepared by INNOTERE GmbH (Radebeul, Germany) as described previously [14]. In brief, a precursor-powder containing 57.3 wt.% alpha-tricalcium phosphate (α-Ca_3_(PO_4_)_2_), 24.8 wt.% calcium hydrogen phosphate (CaHPO_4_), 14.1 wt.% strontium carbonate (SrCO_3_), and 3.8 wt.% hydroxyapatite (Ca_10_(PO_4_)_6_(OH)_2_) was mixed with 2.5 wt.% finely ground K_2_HPO_4_ and dispersed in a non-aqueous carrier liquid of Miglyol 812 with 14.7 wt.% Cremophor ELP and 4.9 wt.% Amphisol A. For preparation of the composite paste pS100G10, MBG particles with a molar ratio of Si/Ca/P = 80/15/5 and with diameters of 100 to 250 µm were synthesized by applying a template-induced, self-assembling method according to Zhu et al. [23] as described previously [25]. The particles were thoroughly dispersed in the pS100 cement paste with a ratio of 10 wt.%. The pS100 and pS100G10 pastes were filled in a syringe and sterilized by gamma-irradiation. After implantation, the cement set within minutes after contact with the aqueous environment [14]. For in vitro investigations of cytocompatibility and ion release/depletion, disc-shaped samples (10 mm diameter, 1 mm height) were prepared from the pastes using silicone molds and allowed to set in water-saturated atmosphere at 37 °C for 3 days.

#### In Vitro Investigations

Cell culture: Human preosteoblasts were isolated from femoral heads of osteoarthritic patients undergoing total hip replacement at the University Hospital Carl Gustav Carus Dresden (Germany) after informed consent (approval by the ethics commission of TU Dresden), as previously described [31]. Cells of three donors in passage 5 were included into the experiments (all female, 52, 75, and 56 years old). Prior to seeding, the cement samples were incubated in 250 µL cell culture medium consisting of Minimal Essential Medium (α-modification, with Glutamax) (α-MEM), 15% fetal bovine serum (Corning), 100 U/mL penicillin, and 100 µg/mL streptomycin for 24 h to allow for medium equilibration. Then, 4 × 10^4^ cells in 250 µL cell culture medium were seeded onto the surface of cement samples. After 24 h of initial cell adhesion, the medium was changed, and osteogenic supplements (10^−7^ M dexamethasone, 10 mM β-glycerophosphate, and 50 µM ascorbic acid 2-phosphate) were included into the medium; samples were cultivated for up to 28 days with a change of medium every 3–4 days. After 1, 7, 14, 21, and 28 days, samples (n = 3 per donor, time point and group) were taken, washed with phosphate-buffered saline (PBS), and frozen at −80 °C until further analysis.

Scanning electron microscopy (SEM): After 21 days of cultivation, cell-seeded samples were fixed with 4% saline-buffered formaldehyde, dehydrated using a series of ethanol with increasing concentrations, and dried using supercritical CO_2_. Samples were mounted on stubs, sputter-coated with carbon and gold, and imaged using a Philips XL 30/ESEM with FEG (field emission gun) operated in SEM mode.

Measurement of DNA content and lactate dehydrogenase (LDH) activity: Cell-seeded samples were thawed, and 1% Triton X-100 in PBS was added. Specimens were incubated for 50 min on ice. During this incubation, samples were sonicated for 10 min in an ice-cold sonification bath. DNA content was quantified from the lysates with Quantifluor dye (Promega, Madison, WI, USA) at an excitation/emission wavelength of 485/535 nm according to manufacturer’s instructions. Cell number was calculated from the DNA content of defined cell numbers. LDH activity was quantified using CytoTox 96^®^ Non-Radioactive Cytotoxicity Assay (Promega). Lysates were diluted 1:5, mixed with assay buffer, and the absorbance at 490 nm was monitored for 5 min. The number of viable cells was calculated from the slope of absorbance using samples with defined cell numbers.

Quantification of ion release and depletion: Cement samples were incubated with 500 µL-supplemented α-MEM resembling cell culture conditions described above. Medium was changed every 3–4 days. The collected medium samples were diluted with 2% nitric acid in deionized water, and the concentrations of calcium, strontium, and silica were quantified using a plasma Quant Elite ICP OES spectrometer (Analytik Jena, Jena, Germany).

### 2.3. Animal Surgery

**Figure 1:**
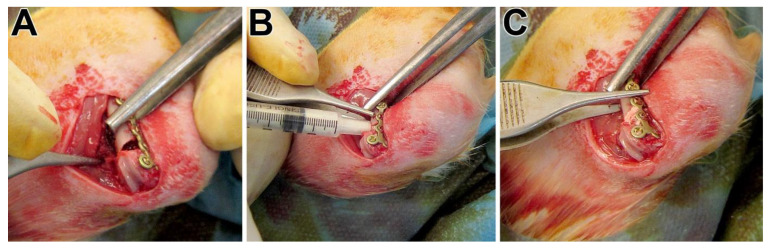
(**A**) Empty defect showing the critical-sized defect; (**B**) injection of the pre-mixed paste (pS100) into the fracture gap using a syringe; (**C**) fracture gap filled with the paste.

We acquired 10-week-old female Sprague–Dawley rats from Charles River (Sulzfeld, Germany). After an acclimatization period of four weeks, the animals were randomly assigned to the three treatment groups. The osteopenic bone status was induced through bilateral ovariectomy by a dorsal approach and a low-calcium, -phosphorous, and -vitamin D3 and soy- and phytoestrogen-free multi-deficient diet (Altromin-C1034, Altromin Spezialfutter GmbH, Lage, Germany) for 12 weeks as described [28,30]. Twelve weeks later, a wedge-shaped fracture-defect with a length of the basis of 4 mm and a medial gap of 0.35 mm at the distal metaphysis of the left femur was created. For this purpose, we used an ultrasound bone saw (Piezosurgery^®^ 3, Saw blade OT7S-3, Mectron, Köln, Germany) with water-cooling. The femur with the fracture-defect was then stabilized by a T-shaped 7-hole mini-plate (Leibinger^®^ XS-miniplate, Stryker, Schönkirchen, Germany), as described by Alt et al. [30]. The created defect was packed either with pS100 or pS100G10 or left empty (Figure 1), corresponding to the randomization. The multi-deficient diet was continually administered until euthanasia after six weeks.

### 2.4. Sample Processing, Staining Procedures, and Histomorphometry

After euthanasia, the femurs were collected and fixed in phosphate-buffered 4% paraformaldehyde for 48 h at 4 °C until processing. They were embedded in Technovit^®^ 9100 NEU according to the manufacturer’s protocol (Heraeus Kulzer, Hanau, Germany). To avoid loss of the implanted biomaterial, we used Kawamoto´s film (Section-Lab Co. Ltd., Yokohama, Japan) when sectioning the embedded Technovit blocks into 5 µm thick slices. The sections were stained with Movat pentachrome and von Kossa/van Gieson stains as described earlier [32,33]. We performed qualitative and quantitative morphological analyses subsequently.

We used the original ROI representing the initial wedge-shaped defect or the histomorphometric analysis of newly formed bone as well as osteoid formation, macrophage count, and implant retention. Adobe Photoshop CS6 was used for the assessments for ROIs, area of bone, implant, osteoid, and the void (sectioning artifacts) to further determine the ratio of bone volume versus tissue volume (BV/TV), osteoid volume versus tissue volume (OV/TV), and implant retention in the defined ROI. The amount of ED1-positive cells per trabecular area (Macrophage/Tb.Ar) was also determined. Assessment was performed blindfolded regarding the different groups. The sequential sections were then used for immunohistochemical and mass spectrometric imaging.

### 2.5. Immunohistochemistry

For immunohistochemical analysis, we used the following antibodies: Rabbit Anti-BMP2 Polyclonal Antibody (AP20597PU-N; Acris, Rockville, MD, USA), Rabbit Anti-Osteoprotegerin Polyclonal Antibody (250800; Abbiotec, Escondido, CA, USA), Rabbit Anti-CD254/RANKL Polyclonal Antibody (AP30826PU-N; Acris, Rockville, MD, USA), Monoclonal Mouse Anti-Human Muscle Actin (M0635; Dako, Santa Clara, CA, USA), Rabbit Anti-Osteocalcin (Dako, Santa Clara, CA, USA), and Mouse Anti-Rat Monocytes/Macrophages Monoclonal Antibody ED1 (MAB1435; Chemicon, Taufkirchen, Germany), correspondingly.

As a secondary antibody for BMP-2, OPG, and RANKL, we used Goat Anti-Rabbit (BA-1000, Vector, Laboratories, Burlingame, CA, USA), followed by a Vectastain ABC kit (Elite PK-6100, Standard, Vector Laboratories, Burlingame, CA, USA). Finally, visualization was performed using Nova Red (SK4800, Vector Laboratories, Burlingame, CA, USA), and hematoxylin (Shandon Inc., Pittburgh, PA, USA) was used as a counterstain. DakoEnvision+System-HRP (DAB) for use with Mouse Primary Antibodies (Dako, Santa Clara, CA, USA, K4006) was utilized for ED1 and ASMA antigen identification.

TRAP staining was performed to analyze osteoclast activity, as described [13] previously. ALP staining was used to analyze the osteoblast activity. The samples were first deplastified and afterwards treated with TRIS buffer of 0.1 molar and pH 9.4. This was followed by incubation with the phosphatase substrate BCIP/NBT (KPL, Gaithersburg, MD, USA) for 2 h at 37 °C. The samples were counterstained with nuclear fast red-aluminum sulfate (Roth, Karlsruhe, Germany).

Images were taken using a Leica DM5500B light microscope with a Leica DCF 7000 T camera (Leica, Wetzlar, Germany) acquired with Leica Application Suite X software (LAS X, Leica Microsystems CMS GmbH, Germany) and administered using Adobe photoshop CS6.

### 2.6. ToF-SIMS Measurements

The surfaces of the 5 µm thick embedded bone sections were deplastified with 2-methoxyethylacetate (MERCK, Darmstadt, Germany). After 3 deplastification steps of 20 min each, the surfaces were suitable for mass spectrometric imaging. Six to eight samples of each group (pS100, pS100G10, empty defect) were analyzed by ToF-SIMS imaging. Large-scale mass spectrometric imaging was carried out on a ToF SIMS 5 instrument from IONTOF GmbH (Münster, Germany). Twenty-five keV Bi_3_^+^ ions were used as primary ion species. The primary ion gun was operated in spectrometry mode to achieve the best possible mass resolution. For charge compensation, low-energy electron flooding was carried out, and analysis took place in cation (positive) mode. To measure sample areas of several mm^2^, we carried out stage scans with the following measurement settings: sawtooths, 10 shots/pixel, 10 frames/patch of the size 400 × 400 µm^2^ and 100 pixels/mm, cycle time 55 µs, 3 scans. The measured primary ion current was 0.5–0.7 pA. The mass spectra were calibrated with the following mass fragments: H^+^, H_2_^+^, CH_3_^+^, Na^+^, CH_4_N^+^, Ca^+^, C_4_H_8_N^+^, and C_5_H_12_N^+^. We achieved a mass resolution of *m*/Δ*m* (FWHM) > 3000 for Ca^+^.

In addition, ToF-SIMS analysis of one bone section of the pS100G10 group was repeated on an M6 Hybrid SIMS instrument (also from IONTOF GmbH) to improve the transmission and lateral as well as mass resolution. On that machine, a 30 kV Bi nanoprobe was mounted, and an additional analyzer lens was used to improve the acceptance angle for the secondary ions (topography mode). Large-area scanning was carried out in sawtooth mode with 3 shots/pixel, 3 frames/patch. Patch size was 400 × 400 µm², pixel density 300 pixels/mm, cycle time 70 µs. In total, 4 scans were carried out in topography mode, with a primary ion current of 0.9 pA. In order to avoid sample charging, we used a low-energy electron source, and in addition, the sample surface was flooded with molecular oxygen via a gas nozzle. The main chamber pressure was kept constant at 5 × 10^−6^ mbar. Internal mass calibration was performed with the following secondary ions: CH_3_^+^, Na^+^, CH_4_N^+^, Ca^+^, C_4_H_8_N^+^, and C_5_H_12_N^+^. Here, too, a mass resolution of better than 3000 was achieved for Ca^+^.

For detailed high-lateral resolution images of the bone cement-filled defect, we also used the M6 instrument. Therefore, a 110 µm beam defining the aperture was used, and imaging with 30 keV Bi_3_^+^ ions was carried out in delayed extraction mode (delay time 150 ns) in positive ion mode. Oxygen gas flooding as well as low energetic electrons were used for charge compensation. Further parameters are listed in Table 1. For internal mass calibration, the following signals were used: Na^+^, CH_4_N^+^, Ca^+^, C_4_H_8_N^+^, and C_5_H_12_N^+^. The achieved mass resolution was *m*/Δ*m* (FWHM) > 3000 for Ca^+^.

For mass spectrometric data evaluation, SurfaceLab 7.1 software from IONTOF GmbH (Münster, Germany) was used. Further experimental and methodic details of mass spectrometric imaging of bone by ToF-SIMS are described in [34,35].

### 2.7. mRNA Preparation and Gene Expression Analysis

We used the following target genes for analysis of the bone metabolism: (A) new bone development: 1. alkaline phosphatase (ALP), an osteoblast marker indicating bone mineralization; 2. osteocalcin (OCN), a non-collagenous protein released by osteoblasts, which plays a role in mineralization and calcium ion homeostasis; 3. collagen type10 alpha1 (Col10α1), a hypertrophic chondrocytes marker; 4. runt-related transcription factor 2 (Runx2), an essential protein for osteoblastic differentiation; 5. collagen type I alpha1, a major component of type I collagen, (Col1α1).

(B) Bone resorption: 1. TNFSF11gene (RANKL, RANK ligand as a member of the tumor necrosis factor (TNF) cytokine family, a ligand for osteoprotegerin and a key factor influencing osteoclast differentiation and activation; 2. TNFRSF11B gene (osteoprotegerin; OPG), a decoy receptor for RANKL by neutralizing its function in osteoclastogenesis; 3. carbonic anhydrase, an osteoclast marker involved in bone matrix dissolution. β2-microglobulin (B2M) was used as a reference gene. The primer pairs are provided in the Supplemental Table S1.

### 2.8. Statistics

Data were tested for normal distribution and homoscedasticity. To analyze the variation between the groups in histomorphometrical analyses, we used one-way ANOVA along with Tukey’s multiple comparison tests. A nonparametric Mann–Whitney U test was used if the requirements were not fulfilled. Statistics were calculated with SPSS V. 20.0 (SPSS Inc., Chicago, IL, USA). The asterisks indicate a significance level of (*) *p* < 0.05, (**) *p* < 0.01, and (***) *p* < 0.001, respectively. Expression analyses were performed by REST © method. *p*-values of less than 0.05 were selected to indicate significance. We used the REST method (based on PCR efficiency and Ct of a sample compared with the control) to determine the relative gene expression ratio for each gene (*E*) and compared it to the reference gene, corresponding to Pfaffl’s mathematical model [36]:

$$ratio=\frac{{\left(E_{target}\right)}^{{∆CP}_{target}(control-sample)}}{{\left(E_{ref}\right)}^{{∆CP}_{ref}(control-sample)}}$$

## 3. Results

### 3.1. In Vitro Characterization of pS100 and pS100G10

**Figure 2:**
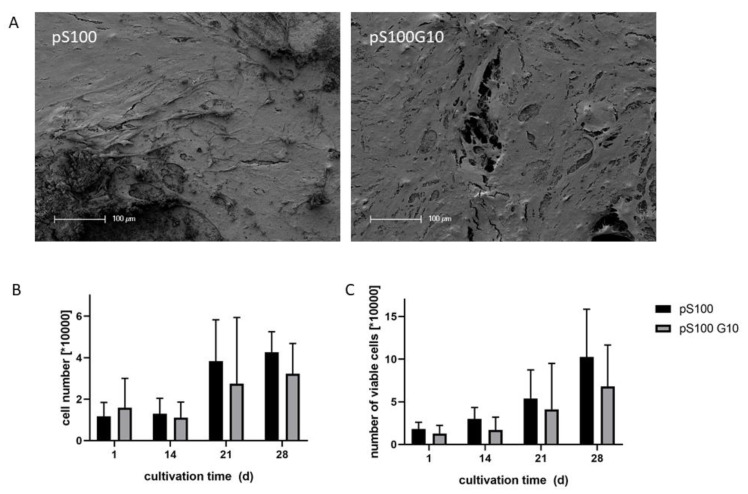
(**A**) SEM images of human osteoblasts on the surface of cement samples after 21 days of cultivation. Scale bars represent 100 µm. (**B**,**C**) Cell number of human osteoblasts after different time points of cultivation. (**B**) Cell number was calculated from DNA content, and (**C**) number of viable cells was calculated from LDH activity after total cell lysis. Data of three different donors are shown, with each *n* = 3 (*n* = 9 in total). Bars represent average +/− standard deviation. Kruskal–Wallis test followed by Dunn’s test for multiple comparisons did not reveal any significant differences between pS100 and pS100G10.

Human pre-osteoblasts proliferated on both cement modifications. SEM investigation revealed a dense cell layer on both pS100 and pS100G10 after 21 days of cultivation (Figure 2A). DNA content of the samples increased during cultivation over 4 weeks (Figure 2B), without statistically significant differences between pS100 and pS100G10. The number of viable cells, analyzed by LDH activity measurement after cell lysis, also increased during cultivation, and no significant differences were shown between both groups (Figure 2C).

**Figure 3:**
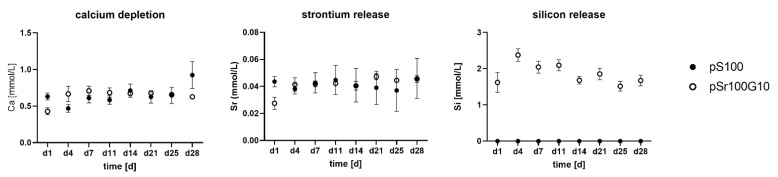
Calcium, strontium, and silicon concentrations in the supernatants of cement samples, incubated in cell culture medium with osteogenic supplements for up to 28 days. Concentrations were analyzed by ICP OES. Average +/− standard deviation (*n* = 3).

Quantification of calcium, strontium, and silicon in the supernatant of cement samples, which was incubated under cell culture conditions, showed a distinct calcium depletion by the samples of both the pS100 and the pS100G10 group. Though the Ca^2+^ concentration of non-treated cell culture medium was around 1.8 mM, its concentration decreased to around 0.5 mM after incubation in the presence of the cement samples (Figure 3A). The addition of MBG did not influence the release of strontium ions from the samples, which was in the same range for both modifications (Figure 3B). The pS100G10 MBG-containing samples showed a steady release of silicon over 4 weeks (Figure 3C).

### 3.2. In Vivo Characterization of pS100 and pS100G10–General Information

At the end of the observation period, 43 of the 45 animals survived. One animal died during the procedure, and one died just after ovariectomy. A total of 15 animals of the empty group, 14 animals of the pS100 group, and 14 animals of the pS100G10 group completed the entire period of record. We did not observe any wound complication or any other serious adverse events.

After explantation of the specimen, we assessed two plate failures in the empty group and one plate failure each in the residual 14 animals in the pS100 and pS100G10 groups. No significant differences were detected for empty defect vs. pS100 and pS100G10 (*p* = 0.5925). These specimens were not taken into further statistical consideration.

### 3.3. Histomorphometry, Histology, and Immunohistochemistry

**Figure 4:**
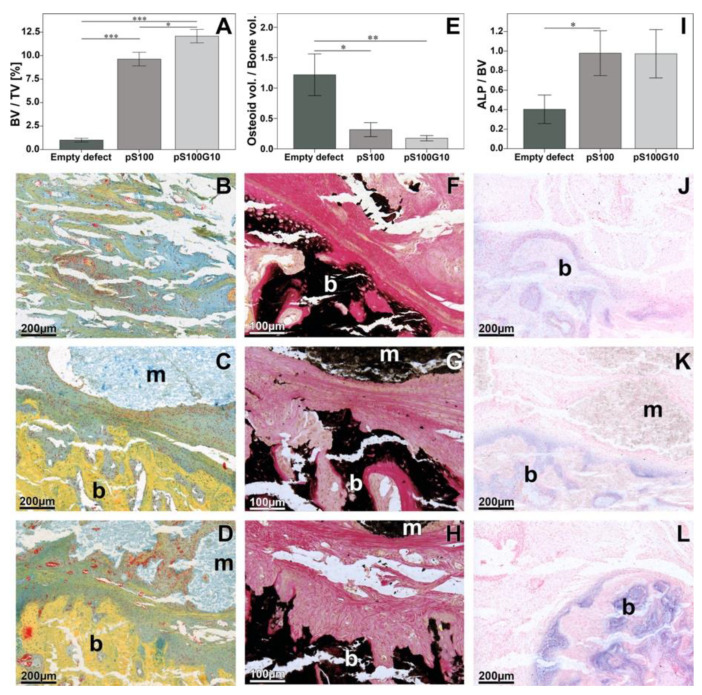
Histomorphometric analysis of bone volume over tissue volume (BV/TV), osteoid volume over tissue volume (OV/TV), and alkaline phosphatase activity over bone volume (ALP/BV) in the first panel ((**A**), * *p* = 0.03, *** *p* = 0.001; (**E**), * *p* = 0.009, ** *p* = 0.004; (**I**), * *p* = 0.03). Histomorphometric analyses reveal an increase in the bone formation in the pS100 and pS100G10 group, with simultaneous decrease in the osteoid volume. Pictorial representation of the tissue sections stained with Movat pentachrome (**B**–**D**) showing the maximum mineralized tissue (yellow) in the pS100 and pS100G10 groups, with von Kossa and van Gieson stains (**F**–**H**), and for alkaline phosphatase activity (**J**–**L**). (**B**,**F**,**J**) Empty defect; (**C**,**G**,**K**) pS100; (**D**,**H**,**L**) S100G10; m = material; b = bone.

Descriptively, an increased biomaterial degradation was seen in the pS100G10 as compared to the pS100 group. Furthermore, the ROI was filled with cell-rich tissue and fibrocartilaginous connective tissue, which was found in the immediate vicinity of the segmented material. Large multinucleated giant cells could also be seen clearly in the pS100G10 group. A larger amount of mineralized tissue (light yellow) was detected in the pS100G10 group compared to the other two groups. Further, an increased chondrocyte and cartilage distribution was seen in the pS100 and pS100G10 groups when compared to the empty group. Furthermore, decreased osteoid formation, depicting better mineralization, was seen in the pS100 and pS00G10 groups as compared to the empty defect alone (Figure 4E–H).

Histomorphometric analysis showed a statistically significant increase in bone formation (BV/TV) in the entire defect region in the pS100G10 group when compared to the pS100 and empty groups (*p* = 0.001), respectively (Figure 4A–D). The utmost bone formation was seen in the pS100G10 group, which was significantly higher when compared to the pS100 group (*p* = 0.03). This increase in bone formation was accompanied by an increase in the alkaline phosphatase activity, which was seen clearly in the test groups when compared to the control group (Figure 4I–L). A simultaneous decrease in osteoid volume (OV/BV) was seen in pS100G10 (*p* = 0.004) and pS100 (*p* = 0.009) compared to the empty defect (Figure 4E–H).

**Figure 5:**
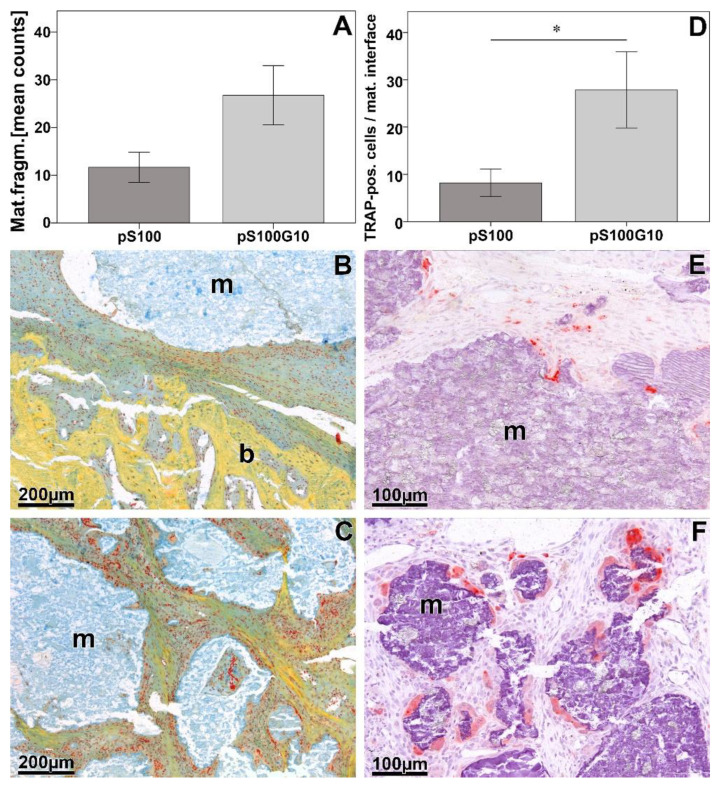
Histomorphometrical analysis of material fragmentation (**A**) and TRAP-positive cells at the material interface (**D**); * *p* < 0.042. Pictorial representation of material fragmentation in pS100 (**B**) and pS100G10 (**C**). TRAP-positive cells at material interface in pS100 (**E**) and pS100G10 (**F**). m = material; b = bone.

Increased material fragmentation was seen in pS100G10 compared to pS100 (*p* = 0.05) (Figure 5A–C). Comparing the number of TRAP-positive cells at the material interface, it was significantly higher in the pS100G10 compared to the pS100 group (*p* = 0.042) (Figure 5D–F).

**Figure 6:**
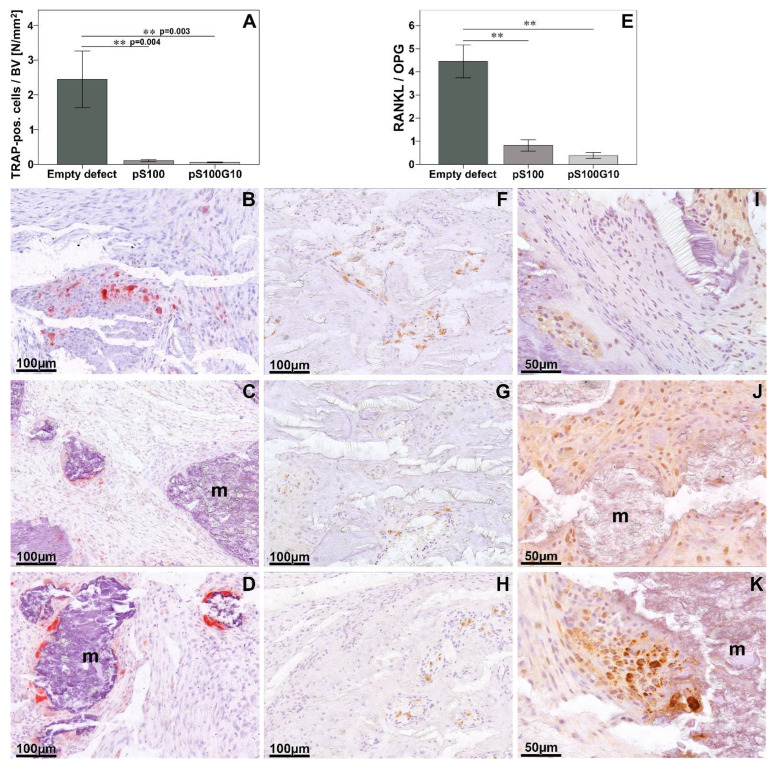
Histomorphometrical analysis of TRAP-positive cells at the bone volume interface (**A**) and RANKL/OPG ratio ((**E**), ** *p* = 0.003 pS100 and *p* = 0.004 pS100G10). Pictorial representation of TRAP-positive cells at bone interface in empty defect (**B**), pS100 (**C**), and pS100G10 (**D**). RANKL- (**F**–**H**) and OPG (**I**–**K**)-positive cells in empty defect, pS100, and pS100G10. m = material.

However, the highest number of TRAP-positive cells was detected in the empty group compared to pS100 (*p* = 0.004) and pS100G10 groups (*p* = 0.003) (Figure 6A). Also, a trend was seen in the increase in ED1-positive cells in the pS100G10 group as compared to the other two groups (*p* = 0.565).

Detailed histology showed that pS100G10 material was very degraded, with small material fragments filling the fracture-defect compared to the pS100 subgroup, in which the material was practically not degraded (Figure 4E). This also facilitated better tissue ingrowth and cell influx in the pS100G10 group compared to the other two groups. Interestingly, we could also see that in some samples, the dissolution of the mesoporous glass allowed for the formation of vessels and tissue ingrowth from within the materials as well (Supplemental Figure S1). pS100G10 exhibited the highest bone formation activity compared to the other two subgroups (Figure 4A–D). There was also an increased chondrocyte and cartilage distribution in pS100G10 and pS100 in comparison to the empty defect (Supplemental Figure S1). Unmineralized osteoid in the defect region was most present in the empty defect group compared to the pS100 and pS100G10 subgroups (Figure 4E–H), which had less osteoid, depicting a better mineralization pattern in the material-substituted groups.

**Figure 7:**
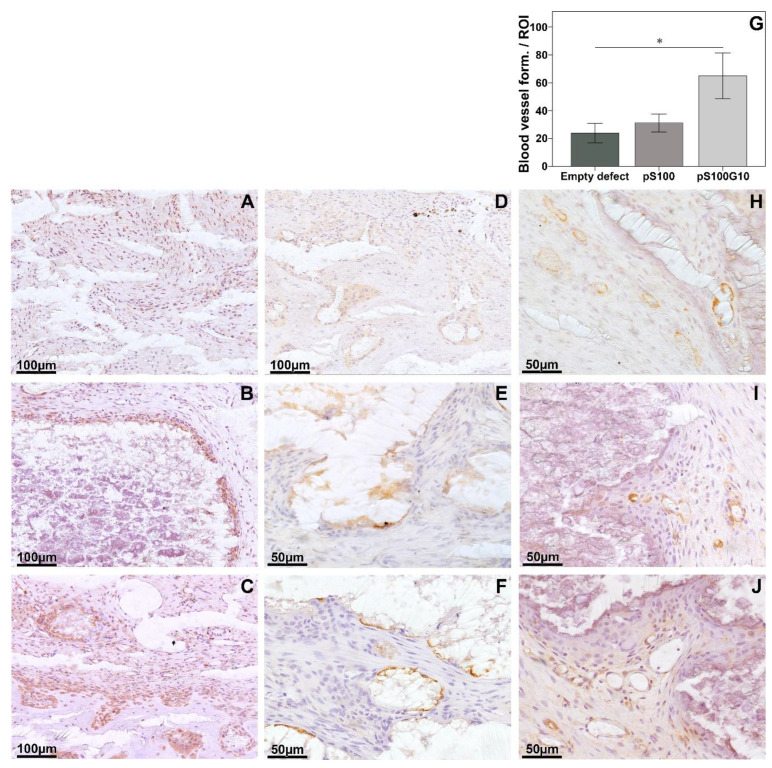
Immunohistochemistry of tissue sections, bone morphogenic protein-2;BMP2 (**A**–**C**), osteocalcin; OCN (**D**–**F**) and alpha smooth muscle actin; αSMA (**H**–**J**)-positive cells both in the entire defect region (left to right: empty, pS100, and pS100G10). An increase in BMP2 and OCN was seen in the pS100 and pS100G10 groups as compared to the empty defect. Immunohistological analysis of blood vessel formation showing the highest blood vessel formation in the pS100G10; * *p* = 0.031 (**G**).

In the immunohistochemical staining, a higher positive bone morphogenic protein-2 expression in the pS100G10 group was followed by a comparatively weaker expression pattern in pS100. There was almost no expression seen in the empty group (Figure 7A–C). A similar trend was seen for the expression of the RANKL/OPG ratio, where the highest ratio was seen in the empty defect group compared to pS100 (*p* = 0.003) and pS100G10 (*p* = 0.004) (Figure 6E). The highest RANKL (receptor activator of nuclear factor kappa-B ligand) expression was also seen in the empty group. This was significantly higher as compared to pS100 (*p* = 0.003) and pS100G10 (*p* = 0.004). The analysis of new blood vessel formation using alpha-smooth muscle actin antibody (Figure 7H–J) revealed a comparatively larger and higher number of positively stained vessels in the pS100G10 group compared to pS100 and empty defect groups, respectively (Figure 7G; *p* = 0.031).

### 3.4. Molecular Biology

**Figure 8:**
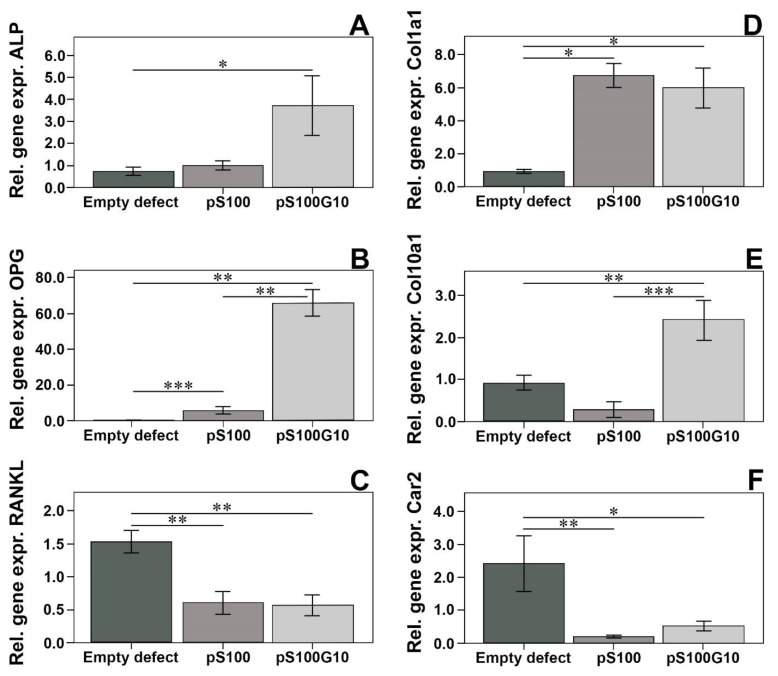
Relative gene expression analysis of bone formation (ALP, * *p* = 0.020 (**A**), OPG, ** *p* = 0.006, *** *p* = 0.003 (**B**), Col1a1 (pS100G10 (*p* = 0.003), pS100 (*p* = 0.001) (**D**), and Col10a1 (** *p* = 0.003, *** *p* = 0.000 (**E**)) and resorption markers (RANKL, ** *p* = 0.004 (**C**), Car2 (* *p* = 0.014, ** *p* = 0.001) (**F**)).

qPCR was used for gene expression analysis. The presented figure shows expression differences between the groups normalized to B2M gene expression (Figure 8). In the pS100G10 group, an enhanced expression of ALP as an osteoblast marker indicating the mineralization of bone was detected compared to the other two groups (*p* = 0.020). Osteoprotegerin, which prevents excessive bone resorption by binding to RANKL, was also significantly higher in the pS100G10 group compared to the pS100 (*p* = 0.006) and empty defect (*p* = 0.003) groups, respectively. A simultaneous reduction in the RANKL expression levels in pS100G10 and pS100 was also seen compared to the empty defect (*p* = 0.004) alone. The expression of Col1a1 was significantly increased in the pS100G10 (*p* = 0.003) and pS100 (*p* = 0.001) groups compared to the empty defect group. No difference in Col1a1 expression was seen between the test groups. In addition, Col10a1 in pS100G10 was significantly higher than that of the pS100 (*p* = 0.000) and empty defect group (*p* = 0.003). Moreover, carbonic anhydrase, an osteoclasts marker involved in bone matrix dissolution, was found to be significantly decreased in pS100G10 (*p* = 0.014) and pS100 (*p* = 0.001) compared to the empty defect group.

### 3.5. ToF-SIMS Analysis

Time-of-flight secondary ion mass spectrometry (ToF-SIMS) is a promising method for bone research. A methodic introduction and its possible applications in bone research was recently published by Kern et al. [37]. Here, mass spectrometric imaging (MSI) was used to investigate the following questions regarding the performance of the biomaterials pS100 and pS100G10. Firstly, MSI analysis was performed to study the release and transport of strontium and silicon from implanted cements into surrounding bone tissue 6 weeks after implantation. For this purpose, ToF-SIMS measurements of bone sections with the biomaterials pS100 and pS100G10 were compared to empty defect bone sections. Furthermore, MSI was used to investigate whether pores were formed in bone sections containing pS100G10 as a biomaterial in direct comparison to sections with pS100 and whether the formation of new bone tissue in these pores takes place.

**Figure 9:**
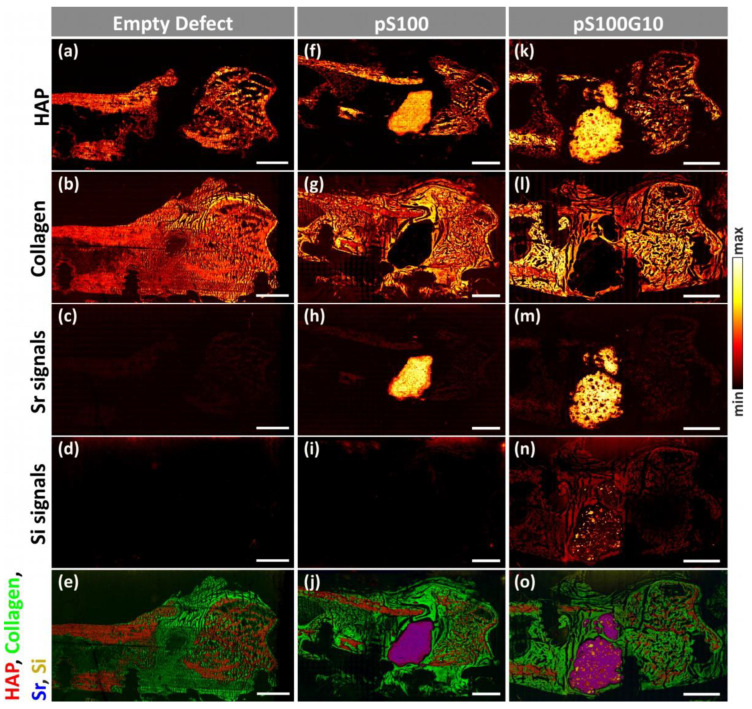
Mass images of rat bone sections containing pS100 (**f**–**j**) and pS100G10 (**k**–**o**) as implanted biomaterials compared to empty defect (**a**–**e**). (**a**,**f**,**k**) For each group, mass signals originating from hydroxyapatite (HAP, signals are listed in Table 2) show mass distribution of HAP in cortical bone, trabecular bone, as well as in remaining bone cements. (**b**,**g**,**l**) Collagen mass images show collagen structure in former defect area, cortical and trabecular bone, as well as in bone marrow. (**c**) Mass imaging of strontium signals (listed in Table 2) shows natural, low-intensity occurrence of strontium in empty defect. For comparison, strontium distribution in bone sections containing biomaterial (**h**,**m**) is shown in remaining bone cement fragments as well as in lower intensity in cortical and trabecular bone. (**d**,**l**,**n**) Mass distribution of silicon signals is only seen in bone sections containing pS100G10 (**n**). The mass image shows glass particles in remains of the pS100G10 cement and the incorporation of silicon in collagenous tissue around former defect area. (**i**,**j**,**o**) Overlay images show mass fragments of mineralized bone in the form of HAP (hydroxyapatite, red), non-mineralized collagen (green), strontium signals (blue), and silicon signals (yellow) (respective mass signals listed in Table 2). (Scale bars = 2 mm).

ToF-SIMS imaging analysis of all three groups clearly shows the spatial arrangement of specific mineralized bone mass fragments in the form of hydroxyapatite (Figure 9a,f,k), collagen (Figure 9b,g,l), strontium signals (Figure 9c,h,m), and silicon signals (Figure 9d,i,n). In overlay images, mass fragments of mineralized bone are depicted in red, collagen mass signals in green, strontium mass signals in blue, and silicon mass signals in yellow (Figure 9e,j,o). A tabular overview of all mass signals used can be found in Table 2.

MSI analysis of bone cross sections with pS100G10 (Figure 9k–o) showed enhanced fragmentation of the biomaterial compared to analysis of the bone sections of the pS100 group (Figure 9f–j). Within the remaining pS100G10 bone cement, mesoporous glass particles could be seen in the form of silicon signals (Figure 9n,o). Moreover, silicon mass signals are visible in the former defect area with the same distribution as collagen, showing the release of silicon from the pS100G10 bone cement as well as the incorporation of silicon into newly formed cartilage tissue. In both the control (Figure 9d) and pS100 (Figure 9i) bone sections, silicon was hardly detectable.

**Figure 10:**
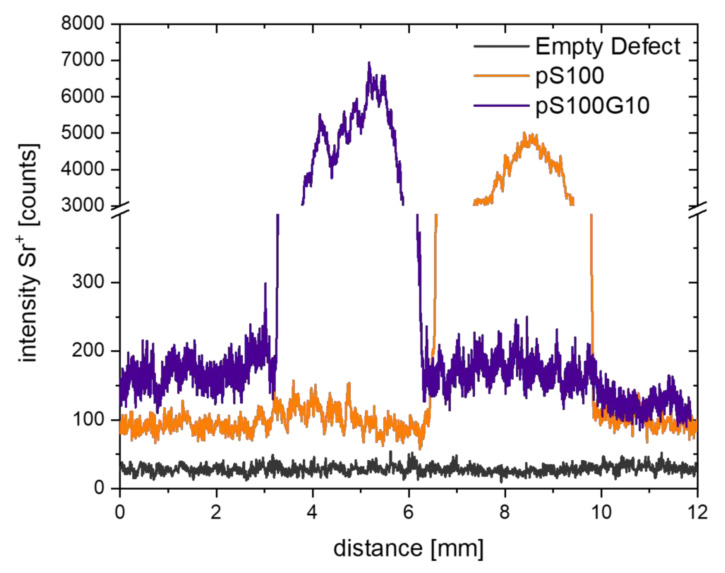
Integral Sr^+^ distribution in bone sections shows intensity gradients from both pS100 (orange) and pS100G10 (blue) biomaterials to enclosing bone tissue. Empty defect (grey) shows natural strontium occurrence in bone with lower intensity and serves as control.

Furthermore, the release and transport of strontium was found in rat bone sections of both the pS100 (Figure 9h) and pS100G10 (Figure 9m) groups in the residual cement fragments, biomaterial–tissue interface regions, as well as in cortical and trabecular bone. Moreover, line scans of Sr^+^ mass images showed intensity gradients from both biomaterials to enclosing bone tissue, which is clear proof of the incorporation of strontium in mineralized bone tissue (Figure 10). The empty defect group served as the control and showed low intensity of natural strontium occurrence (Figure 9c, Figure 10).

**Table 2:** ToF-SIMS analysis with Bi_3_^+^ primary ions as analysis species, performed in positive ion mode, enables detection of mass signals for HAP, collagen, strontium, and silicon in rat bone cross sections of empty defect group as well as groups containing pS100 and pS100G10 as biomaterial. Mass signals of mineralized bone tissue, derived from hydroxyapatite Ca_10_(PO_4_)_6_(OH)_2_, were assigned according to the previous literature [35,38]. Fragments of collagen (amino acids glycine, proline, and hydroxyproline, main components of collagen type 1) were based on collagen peaks known from previous studies [35,38,39]. Mass signals for strontium and silicon were based on mass peaks known from the literature [35].

Mineralized Bone Signals (HAP)				Collagen Signals		Strontium Signals	
*m*/*z*	Peak Label	*m*/*z*	Peak Label	*m*/*z*	Peak Label	*m*/*z*	Peak Label
39.96	Ca^+^	230.78	Ca_3_PO_5_^+^	30.04	CH_4_N^+^	85.89	^86^Sr^+^
55.97	CaO^+^	286.71	Ca_4_PO_6_^+^	44.05	C_2_H_6_N^+^	86.91	^87^Sr^+^
56.96	CaOH^+^			70.06	C_4_H_8_N^+^	87.89	Sr^+^
95.92	Ca_2_O^+^			86.06	C_4_H_8_NO^+^	88.90	SrH^+^
102.92	CaPO_2_^+^					103.90	SrO^+^
111.90	Ca_2_O_2_^+^					104.90	SrOH^+^
112.91	Ca_2_O_2_H^+^					150.85	SrPO_2_^+^
118.92	CaPO_3_^+^					166.85	SrPO_3_^+^
134.88	CaPO_4_^+^					182.86	SrPO_4_^+^
151.84	Ca_3_O_2_^+^			Silicon signals		191.77	Sr_2_O^+^
158.85	Ca_2_PO_3_^+^			*m*/*z*	Peak label	207.80	Sr_2_O_2_^+^
168.84	Ca_3_O_3_H^+^			27.97	Si^+^	208.80	Sr_2_O_2_H^+^
174.84	Ca_2_PO_4_^+^			28.98	SiH^+^	254.80	Sr_2_PO_3_^+^
214.79	Ca_3_PO_4_^+^			44.98	SiOH^+^	270.76	Sr_2_PO_4_^+^

**Figure 11:**
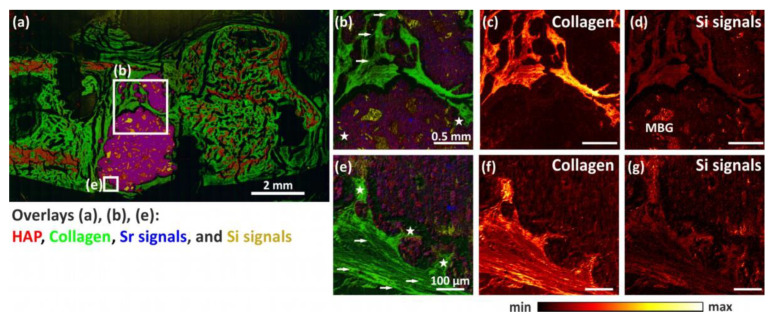
Overview and detailed ToF-SIMS mass images of a bone section containing pS100G10 as biomaterial. Overlay mass images of entire femur bone section (**a**) as well as close-ups of defect area (**b**,**e**) show mineralized bone (HAP) in red, collagen in green, strontium (blue), and silicon signals (yellow) (mass signals are listed in Table 2). (**b**,**e**) Close-ups of defect area show pore formation in remaining cement fragments with newly formed collagenous bone tissue within these pores (indicated with a white star *). Collagen fibrils are indicated with white arrows. (**c**,**f**) Mass images show distribution of collagen. (**d**,**g**) High-resolution mass images show distribution of silicon signals in the same area as collagen and intact mesoporous glass particles (MBG) within the bone cement. Detailed measurement conditions for (**b**–**g**) are given in Table 1.

The overlay mass image (Figure 10a) of a pS100G10-containing rat femur cross-section shows the distribution of HAP in red, collagen in green, strontium signals in blue, and silicon signals in yellow (signals listed in Table 2). High-resolution ToF-SIMS mass images enable a detailed study of the former defect area around the fragmented bone cement (Figure 11b–g). Mass images of silicon signals (Figure 11d,g) show the silicon mass distribution in the initially created defect area in the same areas as those in which collagen occurs (Figure 11c,f). This is clear evidence that silicon is incorporated into newly formed collagenous soft bone tissue. Additionally, intact mesoporous bioactive glass particles (MBG) within bone cement could be detected (Figure 11d). Furthermore, pores were seen in the biomaterial, preliminarily at the cement–tissue interface. Due to contact with body fluids or bone marrow, the MBG obviously dissolved as expected (Figure 11b,e; indicated with a white star). Within these pores, newly formed bone tissue could be proven in the form of collagen mass signals (Figure 11b,c,e,f) This could also be validated by the histological findings and further supports the histomorphometric analysis. Furthermore, we were able to visualize the fibril structure of fresh collagenous tissue within the former defect area (indicated by arrows in Figure 11b,e), surrounding the bone cement.

**Figure 12:**
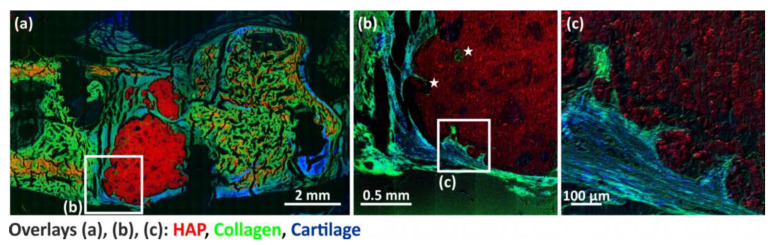
Overview and detailed mass images of pS100G10-containing bone sections. RGB overlay images show mass distribution of HAP (mineralized bone, red), collagen mass signals (green) (listed in Table 2), as well as signals related to cartilage (sum of Na_3_SO_4_^+^ and Na_5_S_2_O_8_^+^ sulfate signals) in blue. (**a**) Cartilage tissue in the form of sulfate signals (blue) is shown in areas of newly formed bone and growth joint. (**b**,**c**) High-resolution detailed mass images show sulfate signals (blue) in areas of newly formed collagenous tissue (green) within the collagen matrix. Newly formed collagenous bone tissue was also detected in pores within biomaterial (indicated with a white star *). Detailed measurement conditions for (**b**,**c**) are given in Table 1.

In addition, newly formed bone tissue in the form of sulfate signals, most likely originating from proteoglycans, according to [35], could also be detected in areas of the former defect (Figure 12 An overview mass image of the pS100G10-containing bone section (Figure 12a) shows mineralized areas in red, collagen-related signals in green, and sulfate signals in blue. Sulfate signals occur in areas of newly formed bone (more visible in detailed images in Figure 12b,c), cartilage tissue, and growth joint. High-resolution mass images (delayed extraction mode; Figure 12b,c) of bone cement–bone tissue interface in the former defect area revealed that sulfate signals (blue) are found in areas of newly formed bone within the collagen matrix (green).

## 4. Discussion

The aim of the present study was to investigate the bone formation behavior of a strontium(II)-modified, injectable calcium phosphate cement containing mesoporous bioactive glass particles (“pS100G10”) in comparison to a plain version of the strontium(II)-modified calcium phosphate cement (“pS100”) after implantation into an osteoporotic defect model in rats.

In vitro cytocompatibility of the “pS100G10” CPC-MBG composite was proven by direct seeding of human preosteoblasts obtained from three donors. The cells were able to attach themselves to the sample surface and to proliferate over the culture period of 28 days, without significant difference between pS100G10 and pS100. Previously, significantly higher metabolic activity was observed for the osteoblast-like cell line SAOS-2 when cultured on composites of a conventional powder/liquid CPC and 10 wt% MBG vs. pure powder/liquid CPC samples. However, in that study, the CPC was not strontium-modified [25]. Quantification of calcium, strontium, and silicon in the supernatant, reflecting the exchange of ions between the material samples and the cell culture medium, indicated a significant difference between pS100G10 and pS100 only for silicon ions that were released from the silicate-based MBG particles in pS100G10 only. The measured concentration was around 2 mmol/L and was therefore in a range that is known to be compatible with osteoblasts and even capable of stimulating osteoblast differentiation [40,41,42]. For both materials, a decrease in the calcium ion concentration of the cell culture medium was measured, which confirmed our previous observations for the pS100 and the strontium-free counterpart pCPC [14] as well as for the powder/liquid-type cements [11]. No significant impact of the integrated MBG particles was observed in the present study. This calcium depletion can be explained by the bioactivity of α-tricalcium phosphate-based CPCs, i.e., the formation of calcium phosphate crystals on the material surface upon immersion in serum-like liquids [43]. The constant release of strontium ions over time, detected for both pS100G10 and pS100 without significant difference and with around 0.04 mmol/L in the biologically effective concentration range [11], might have counteracted the negative effects of calcium depletion on cellular functions like proliferation, as suggested previously [14]. Altogether, the results of the in vitro characterization indicated no significant effect of the integration of 10 wt% MBG in pS100 on cytocompatibility and the release of strontium ions.

The release of silicon ions suggests an ongoing dissolution of the MBG component, which might result in the formation of voids in the bulk cement matrix, as observed in vitro in earlier studies for a comparable powder/liquid-based CPC-MBG composite as well as for pS100G10 [25,26]. Moreover, a significant increase in porosity as well as a significantly higher mass loss during incubation were measured previously when CPC-MBG composite scaffolds were compared to pure CPC scaffolds, which again indicated dissolution of the MBG component [25,44]. These in vitro findings are in line with the observation of a significantly increased material degradation of pS100G10 compared to pS100 in vivo. ToF-SIMS analysis of explants showed that the degradation of the MBG particles released silicon ions, which were incorporated into newly formed bone tissue at the bone tissue–implant interface. Silicon is an essential trace element, present in the bone tissue and required for physiological bone development [45]. Based on observations after dietary intake in chicken and humans [46,47], a positive effect of the silicon ions, released from the MBG particles, on bone formation and mineralization is to be assumed. This assumption is underlined by other in vivo studies demonstrating significantly increased bone formation by silicon substitution of calcium phosphate bioceramics [48,49] or by silicate-based bioactive glasses compared to the respective negative controls [50].

The increased material degradation by MBG dissolution led to fragmentation of the implant, which is expected to not only allow for an enhanced ingrowth of new bone tissue but also to enhance the release of strontium ions from the cement matrix in vivo due to the higher surface-to-volume ratio. This assumption is supported by a recent study of strontium release from 3D-plotted macroporous scaffolds of pS100 with and without integrated MBG particles. Although the integration of strontium-free MBG into pS100 reduces the total amount of strontium ions that can theoretically be released from the composites, a higher release was observed for the composites compared to pure pS100 scaffolds. This can be attributed to the faster degradation of the MBG particles, which leads to an increased surface area, and thus an improved degradation of the pS100 cement [44].

Histologically, both pS100 and pS100G10 showed increased new bone formation compared to the empty defect group. In addition, the MBG-containing group pS100G10 exhibited more developed bone tissue, shown by increased mineralized and less osteoid tissue than the plain cement pS100 group and the empty defect group. Also, quantitative analysis of the newly formed bone showed increased mineralized bone tissue in the MBG-containing material group pS100G10. This is in line with the abovementioned expectation that the released silicon ions may have a positive effect on bone formation and mineralization. A possibly increased release of strontium ions could also have contributed to this. The histological data were supported by immunohistochemical staining of BMP-2 as well as by gene expression analysis of ALP and Col10a1, which indicated a significantly higher expression of osteoblastic markers in the pS100G10 group vs. the pS100 group.

Enzyme-histochemical analysis revealed an increased amount of multinucleated giant cells in the MBG-containing group pS100G10, suggesting an increase in the tissue turnover and material degradation of the composite compared to the plain pS100. Increased bone remodeling at the material–tissue interface was also described after the implantation of silicon-substituted hydroxyapatite granules or scaffolds in an ovine or rabbit model, respectively [48,51]; silicon has been described to support both bone-resorbing and bone-forming cells [52,53], and its level seemed to be a determinant for the net amount of bone ingrowth [48]. Recently, Wagner et al. investigated the osteoclastogenesis of human peripheral blood mononuclear cells (PBMC) in extracts obtained from pS100G10 (containing both strontium and silicon ions) and pS100 (containing strontium ions only) and observed significantly improved PBMC fusion as well significantly increased expression of fusion and osteoclastic markers in the pS100G10 extracts [26]. This confirms an effect of silicon on osteoclast formation and indicates that the presence of silicon may alter the effect of strontium on osteoclasts, which is known from a previous in vitro study investigating the powder-liquid strontium-modified CPC “S100” to reduce their resorbing activity [12].

The RANKL-to-OPG relation is an important determinant of bone remodeling and the protection of bone mass. Any alternation in this ratio has been confirmed in several bone loss-associated diseases. We detected an elevated OPG and decreased RANKL value in the pS100 group and especially in the MBG-containing pS100G10 group. A significant difference was also found for the RANKL/OPG ratio, as revealed by the immunohistochemistry data. We confirmed that osteoclast activity is likely to depend on the relative balance of RANKL and OPG, which is the critical factor for determining osteoclastic activation at the bone level. Regarding the RANKL/OPG ratio, both strontium-modified cements, pS100 and pS100G10, showed a comparable performance in contrast to the empty defect.

An essential prerequisite for better osseointegration and healing of the bone defect is a fast vascularization of the defect region. Immunohistological analysis revealed the best blood vessel formation, indicated by the size and number of ASMA-positive stained vessels, in the pS100G10 group. There are a number of studies demonstrating the pro-angiogenic effect of bioactive glasses, which is mediated by their ionic dissolution products [50,54,55]. The specific effect of silicon ions in stimulating angiogenesis, e.g., via up-regulation of the expression of the angiogenic factors VEGF or bFGF, has been demonstrated [56,57]. However, the strontium ions released from the cement component, possibly enhanced in the pS100G10 group, as discussed above, could also have supported blood vessel formation in the defect region, as strontium ions were shown to enhance the secretion of angiogenic factors (VEGF, angiogenin-1) in endothelial cell monocultures and co-cultures with bone marrow-derived mesenchymal stromal cells as well as tube formation [58]. Consistently, in our previous study, increased CD31 expression was detected at the tissue–implant interface of the powder/liquid strontium-modified CPC “S100” in comparison to the strontium-free CPC and the empty control group [13]. Finally, the pore formation and enhanced fragmentation of the material in the pS100G10 group could have provided more space for blood vessel formation.

By using ToF-SIMS analysis, we could prove that dissolved MBG was substituted by the in-growth of collagenous tissue. We were able to show this in the form of collagen mass signals that were found within these pores. These results are in line with our previous study [35].

In addition, as shown in our previous study [35], we detected newly formed bone tissue in the form of sulfate signals, most likely originating from proteoglycans, within the collagen matrix inside the defect region. Despite better biological integration of the MBG-CPC in the defect region, this material acts only as an osteoconductive matrix. In a previous study, we could show that strontium-functionalized CPC (SrCPC) had a much better performance in new bone formation than a plain calcium phosphate cement [13].

Furthermore, we could prove that the integration of strontium ions is not only confined to the newly formed bone tissue but is also found within the entire defect region. As in the SrCPC study, we performed ToF-SIMS analysis to show the release of strontium ions from the biomaterials pS100 and pS100G10 into the surrounding tissue. In both groups, we detected Sr ions not only in the remains of biomaterials but also in mineralized bone tissue in cortical and trabecular bone areas, indicating the incorporation of strontium in bone. These findings are in line with previous ToF-SIMS studies of bone sections containing pS100 and pS100G10 cement [59,60]. This is again a proof of the principle that strontium ions could be released by a different carrier, serving as a substitute bone material and giving them an osteoinductive feature [61].

Another study investigated the in vivo performance of 3D-plotted, macroporous scaffolds consisting of the strontium-modified CPC “pS100” in comparison to strontium-free pCPC after implantation into unloaded as well as load-bearing bone defects in healthy sheep. The positive effect of the strontium modification was confirmed, as significantly improved new bone formation for the strontium-modified cement group was observed. The macroporous structure, generated by the 3D-plotting process, also enabled fast ingrowth of tissue [62]. Composites of the pasty CPC, including pS100, with different amounts of MBG (up to 16 wt%) have also been successfully applied for 3D plotting of macroporous scaffolds, which opens up the possibility to fabricate implants with defect-adapted geometry and to tailor the internal structure/macroporosity [63]. The mesoporous structure of the MBG allows for the integration of proteins and growth factors prior to integration into the CPC matrix—as exemplarily shown for lysozyme and VEGF—which can further support bone regeneration [25,44]. The results of the present study are furthermore in line with a previous work investigating bioglass microspheres incorporated into a brushite cement: better bone formation in the brushite-bioglass composite compared to a plain brushite group as well faster degradation of the composite material were observed [64]. The authors drew the same conclusion that the developing pores in the material, induced by the degradation of the bioglass, enhanced the tissue ingrowth.

The aim of in vivo animal studies should be a simulation of the human situation in the most comparable manner to gain highly predictable information. Thus, animal fracture models must consider the issue of a complete disruption of the bone, and the method must be highly reproducible [30]. Most of the existing studies use only drill hole defects, e.g., [64], and they do not simulate the biomechanical demands of an instable fracture. In the current study, we used a metaphyseal fracture defect model, which allows us to investigate different biomaterials in a clinically relevant fracture defect, stabilized with a T-shaped plate on the distal femur, like in the human situation [30]. Ovariectomy and a special diet were shown to lead to a significant reduction in bone mineral density compared to sham animals [27,28,29]. Thus, the used biomaterials have shown their potential in supporting bone defect healing under systemically impaired bone metabolism and in a more realistic defect model. As a drawback, we saw two plate failures in the control group and one in each material group. Indeed, no statistically relevant difference was found, but there was a trend towards the empty defect, suggesting that the biomechanical stress on the osteosynthesis material is less in the materials groups. This reflects the human situation, in which we also see plate failure if the bone healing does not overrun the material fatigue and leads to a plate breakage. To reduce bias, we excluded these groups from further analysis.

## 5. Conclusions

The mesoporous bioactive glass modification of the injectable pasty strontium-containing calcium phosphate bone cement resulted in significantly higher new bone formation compared to the bioglass-free strontium-calcium phosphate bone cement and the empty defect in a critical-sized metaphyseal bone defect model in osteopenic rats. Key bone formation markers, such as the RANKL/OPG ratio and BMP2, were significantly enhanced in the mesoporous bioglass group compared to the two other groups. ToF-SIMS imaging also revealed that resorption of the MBG particles allowed for new bone formation in cement pores. Mass spectrometric imaging by time-of-flight secondary ion mass spectrometry (ToF-SIMS) showed the release of Sr^2+^ ions from both pS100 and pS100G10, with a gradient into the interface region. The addition of MBG led to improved degradation of the implant material; the faster dissolution of the MBG resulted in pore formation within and fragmentation of the cement matrix. This facilitates blood vessel formation and accelerates tissue ingrowth, leading to a higher amount of newly formed bone. Thus, the combination of enhanced degradation with increasing porosity, together with the bone-stimulating effect of strontium, makes the “pS100G10” CPC-MBG composite a promising bone substitute material for the treatment of large fracture-defects in systemically compromised bone.

## Figures and Tables

**Table 1:** Parameters for detailed ToF-SIMS images measured in cation mode with M6 Hybrid SIMS (IONTOF GmbH, Münster, Germany).

Figure	11b–d	11e–g, 12c	12b
Analysis options			
Cycle time	65 µs	65 µs	65 µs
Analysis area	2 × 2 mm^2^	500 × 500 µm^2^	2 × 2 mm^2^
Raster mode	sawtooth	sawtooth	sawtooth
Primary ion current	0.05 pA	0.04 pA	0.05 pA
Pixel resolution	1600 × 1600	1024 × 1024	2000 × 2000
Frames per patch	3	1	3
Patch size	0.4 mm	0.5 mm	0.4 mm
Primary ion Shots/frame/pixel	3	2	3
Number of scans	10	150	15

## Data Availability

The raw/processed data required to reproduce these findings cannot be shared at this time, as the data also form part of an ongoing study.

## Supplementary Materials

The following supporting information can be downloaded at: https://www.mdpi.com/article/10.3390/bioengineering10101203/s1, Figure S1: Dissolution of the mesoporous glass allowed the formation of vessels and tissue ingrowth. Table S1: Primer pairs.

## References

[1] Wang P., Zhao L., Chen W., Liu X., Weir M.D., Xu H.H. (2014). Stem Cells and Calcium Phosphate Cement Scaffolds for Bone Regeneration. J. Dent. Res..

[2] Dorozhkin S.V. (2011). Self-Setting Calcium Orthophosphate Formulations: Cements, Concretes, Pastes and Putties. Int. J. Mater. Chem..

[3] Heinemann S., Rossler S., Lemm M., Ruhnow M., Nies B. (2013). Properties of injectable ready-to-use calcium phosphate cement based on water-immiscible liquid. Acta Biomater..

[4] Bohner M., Tiainen H., Michel P., Döbelin N. (2015). Design of an inorganic dual-paste apatite cement using cation exchange. J. Mater. Sci. Mater. Med..

[5] Habibovic P., Barralet J.E. (2011). Bioinorganics and biomaterials: Bone repair. Acta Biomater..

[6] Yang F., Yang D., Tu J., Zheng Q., Cai L., Wang L. (2011). Strontium enhances osteogenic differentiation of mesenchymal stem cells and in vivo bone formation by activating Wnt/catenin signaling. Stem Cells.

[7] Barbara A., Delannoy P., Denis B.G., Marie P.J. (2004). Normal matrix mineralization induced by strontium ranelate in MC3T3-E1 osteogenic cells. Metabolism.

[8] Bonnelye E., Chabadel A., Saltel F., Jurdic P. (2008). Dual effect of strontium ranelate: Stimulation of osteoblast differentiation and inhibition of osteoclast formation and resorption in vitro. Bone.

[9] Schumacher M., Gelinsky M. (2015). Strontium modified calcium phosphate cements—Approaches towards targeted stimulation of bone turnover. J. Mater. Chem. B.

[10] Schumacher M., Henß A., Rohnke M., Gelinsky M. (2013). A novel and easy-to-prepare strontium(II) modified calcium phosphate bone cement with enhanced mechanical properties. Acta Biomater..

[11] Schumacher M., Lode A., Helth A., Gelinsky M. (2013). A novel strontium(II)-modified calcium phosphate bone cement stimulates human-bone-marrow-derived mesenchymal stem cell proliferation and osteogenic differentiation in vitro. Acta Biomater..

[12] Schumacher M., Wagner A.S., Kokesch-Himmelreich J., Bernhardt A., Rohnke M., Wenisch S., Gelinsky M. (2016). Strontium substitution in apatitic CaP cements effectively attenuates osteoclastic resorption but does not inhibit osteoclastogenesis. Acta Biomater..

[13] Thormann U., Ray S., Sommer U., Elkhassawna T., Rehling T., Hundgeburth M., Henß A., Rohnke M., Janek J., Lips K.S. (2013). Bone formation induced by strontium modified calcium phosphate cement in critical-size metaphyseal fracture defects in ovariectomized rats. Biomaterials.

[14] Lode A., Heiss C., Knapp G., Thomas J., Nies B., Gelinsky M., Schumacher M. (2018). Strontium-modified premixed calcium phosphate cements for the therapy of osteoporotic bone defects. Acta Biomater..

[15] Bernhardt A., Schumacher M., Gelinsky M. (2015). Formation of osteoclasts on calcium phosphate bone cements and polystyrene depends on monocyte isolation conditions. Tissue Eng. Part C Methods.

[16] Felix Lanao R.P., Leeuwenburgh S.C., Wolke J.G., Jansen J.A. (2011). In vitro degradation rate of apatitic calcium phosphate cement with incorporated PLGA microspheres. Acta Biomater..

[17] Felix Lanao R.P., Leeuwenburgh S.C., Wolke J.G., Jansen J.A. (2011). Bone response to fast-degrading, injectable calcium phosphate cements containing PLGA microparticles. Biomaterials.

[18] Lodoso-Torrecilla I., Stumpel F., Jansen J., van den Beucken J. (2020). Early-stage macroporosity enhancement in calcium phosphate cements by inclusion of poly(N-vinylpyrrolidone) particles as a porogen. Mater. Today Commun..

[19] Jones J.R. (2015). Reprint of: Review of bioactive glass: From Hench to hybrids. Acta Biomater..

[20] Hench L.L., Jones J.R. (2015). Bioactive Glasses: Frontiers and Challenges. Front. Bioeng. Biotechnol..

[21] Bosetti M., Cannas M. (2005). The effect of bioactive glasses on bone marrow stromal cells differentiation. Biomaterials.

[22] Hoppe A., Guldal N.S., Boccaccini A.R. (2011). A review of the biological response to ionic dissolution products from bioactive glasses and glass-ceramics. Biomaterials.

[23] Zhu Y., Wu C., Ramaswamy Y., Kockrick E., Simon P., Kaskel S., Zreiqat H. (2008). Preparation, characterization and in vitro bioactivity of mesoporous bioactive glasses (MBGs) scaffolds for bone tissue engineering. Microporous Mesoporous Mater..

[24] Wu C., Chang J. (2012). Mesoporous bioactive glasses: Structure characteristics, drug/growth factor delivery and bone regeneration application. Interface Focus.

[25] Schumacher M., Reither L., Thomas J., Kampschulte M., Gbureck U., Lode A., Gelinsky M. (2017). Calcium phosphate bone cement/mesoporous bioactive glass composites for controlled growth factor delivery. Biomater. Sci..

[26] Wagner A., Schumacher M., Rohnke M., Glenske K., Gelinsky M., Arnhold S., Mazurek S., Wenisch S. (2019). Incorporation of silicon into strontium modified calcium phosphate bone cements promotes osteoclastogenesis of human peripheral mononuclear blood cells. Biomed. Mater..

[27] Govindarajan P., Schlewitz G., Schliefke N., Weisweiler D., Alt V., Thormann U., Lips K.S., Wenisch S., Langheinrich A.C., Zahner D. (2013). Implications of combined ovariectomy/multi-deficiency diet on rat bone with age-related variation in bone parameters and bone loss at multiple skeletal sites by DEXA. Med. Sci. Monit. Basic Res..

[28] Heiss C., Govindarajan P., Schlewitz G., Hemdan N.Y.A., Schliefke N., Alt V., Thormann U., Lips K.S., Wenisch S., Langheinrich A.C. (2012). Induction of osteoporosis with its influence on osteoporotic determinants and their interrelationships in rats by DEXA. Med. Sci. Monit..

[29] Schlewitz G., Govindarajan P., Schliefke N., Alt V., Böcker W., Elkhassawna T., Thormann U., Lips K.S., Hemdan N.Y., Zahner D. (2013). Ovariectomy and calcium/vitamin D2/D3 deficient diet as a model of osteoporosis in the spine of Sprague-Dawley rats. Z. Orthop. Unf..

[30] Alt V., Thormann U., Ray S., Zahner D., Dürselen L., Lips K., El Khassawna T., Heiss C., Riedrich A., Schlewitz G. (2013). A new metaphyseal bone defect model in osteoporotic rats to study biomaterials for the enhancement of bone healing in osteoporotic fractures. Acta Biomater..

[31] Bernhardt A., Wolf S., Weiser E., Vater C., Gelinsky M. (2020). An improved method to isolate primary human osteocytes from bone. Biomed. Eng..

[32] Albers J., Schulze J., Beil F.T., Gebauer M., Baranowsky A., Keller J., Marshall R.P., Wintges K., Friedrich F.W., Priemel M. (2011). Control of bone formation by the serpentine receptor Frizzled-9. J. Cell Biol..

[33] Peters A., Toben D., Lienau J., Schell H., Bail H.J., Matziolis G., Duda G.N., Kaspar K. (2009). Locally applied osteogenic predifferentiated progenitor cells are more effective than undifferentiated mesenchymal stem cells in the treatment of delayed bone healing. Tissue Eng. Part A.

[34] Henss A., Rohnke M., El Khassawna T., Govindarajan P., Schlewitz G., Heiss C., Janek J. (2013). Applicability of ToF-SIMS for monitoring compositional changes in bone in a long-term animal model. J. R. Soc. Interface.

[35] Kern C., Ray S., Gelinsky M., Bellew A., Pirkl A., Rohnke M. (2020). New insights into ToF-SIMS imaging in osteoporotic bone research. Biointerphases.

[36] Pfaffl M.W. (2001). A new mathematical model for relative quantification in real-time RT-PCR. Nucleic Acids Res..

[37] Kern C., Kern S., Henss A., Rohnke M. (2023). Secondary ion mass spectrometry for bone research. Biointerphases.

[38] Henss A., Hild A., Rohnke M., Wenisch S., Janek J. (2016). Time of flight secondary ion mass spectrometry of bone—Impact of sample preparation and measurement conditions. Biointerphases.

[39] Kokesch-Himmelreich J., Schumacher M., Rohnke M., Gelinsky M., Janek J. (2013). ToF-SIMS analysis of osteoblast-like cells and their mineralized extracellular matrix on strontium enriched bone cements. Biointerphases.

[40] Valerio P., Pereira M.M., Goes A.M., Leite M.F. (2004). The effect of ionic products from bioactive glass dissolution on osteoblast proliferation and collagen production. Biomaterials.

[41] Zhou H., Wu X., Wei J., Lu X., Zhang S., Shi J., Liu C. (2011). Stimulated osteoblastic proliferation by mesoporous silica xerogel with high specific surface area. J. Mater. Sci. Mater. Med..

[42] Shie M.Y., Ding S.J., Chang H.C. (2011). The role of silicon in osteoblast-like cell proliferation and apoptosis. Acta Biomater..

[43] Bohner M., Lemaitre J. (2009). Can bioactivity be tested in vitro with SBF solution?. Biomaterials.

[44] Richter R.F., Ahlfeld T., Gelinsky M., Lode A. (2022). Composites consisting of calcium phosphate cements and mesoporous bioactive glasses as a 3D plottable drug delivery system. Acta Biomater..

[45] Carlisle E.M. (1972). Silicon: An essential element for the chick. Science.

[46] Carlisle E.M. (1981). Silicon: A requirement in bone formation independent of vitamin D1. Calcif. Tissue Int..

[47] Jugdaohsingh R., Tucker K., Cupples N., Kiel D., Powell J. (2004). Dietary Silicon Intake is Positively Associated with Bone Mineral Density in Men and Premenopausal Women of the Framingham Offspring Cohort. JBMR.

[48] Hing K.A., Revell P.A., Smith N., Buckland T. (2006). Effect of silicon level on rate, quality and progression of bone healing within silicate-substituted porous hydroxyapatite scaffolds. Biomaterials.

[49] Patel N., Best S.M., Bonfield W., Gibson I.R., Hing K.A., Damien E., Revell P.A. (2002). A comparative study on the in vivo behavior of hydroxyapatite and silicon substituted hydroxyapatite granules. J. Mater. Sci. Mater. Med..

[50] Ranmuthu C.D.S., Ranmuthu C.K.I., Russell J.C., Singhania D., Khan W.S. (2020). Evaluating the Effect of Non-cellular Bioactive Glass-Containing Scaffolds on Osteogenesis and Angiogenesis in in vivo Animal Bone Defect Models. Front. Bioeng. Biotechnol..

[51] Porter A.E., Patel N., Skepper J.N., Best S.M., Bonfield W. (2004). Effect of sintered silicate-substituted hydroxyapatite on remodelling processes at the bone-implant interface. Biomaterials.

[52] Botelho C.M., Brooks R.A., Spence G., McFarlane I., Lopes M.A., Best S.M., Santos J.D., Rushton N., Bonfield W. (2006). Differentiation of mononuclear precursors into osteoclasts on the surface of Si-substituted hydroxyapatite. J. Biomed. Mater. Res. A.

[53] Botelho C.M., Brooks R.A., Best S.M., Lopes M.A., Santos J.D., Rushton N., Bonfield W. (2006). Human osteoblast response to silicon-substituted hydroxyapatite. J. Biomed. Mater. Res. A.

[54] Gorustovich A.A., Roether J.A., Boccaccini A.R. (2010). Effect of bioactive glasses on angiogenesis: A review of in vitro and in vivo evidences. Tissue Eng. Part B Rev..

[55] Hoppe A., Boccaccini A.R. (2015). Biological Impact of Bioactive Glasses and Their Dissolution Products. Front. Oral Biol..

[56] Li H., Chang J. (2013). Stimulation of proangiogenesis by calcium silicate bioactive ceramic. Acta Biomater..

[57] Li H., Chang J. (2013). Bioactive silicate materials stimulate angiogenesis in fibroblast and endothelial cell co-culture system through paracrine effect. Acta Biomater..

[58] Yan R., Li J., Wu Q., Zhang X., Hu L., Deng Y., Jiang R., Wen J., Jiang X. (2022). Trace Element-Augmented Titanium Implant With Targeted Angiogenesis and Enhanced Osseointegration in Osteoporotic Rats. Front. Chem..

[59] Kern C., Quade M., Ray S., Thomas J., Schumacher M., Gemming T., Gelinsky M., Alt V., Rohnke M. (2019). Investigation of strontium transport and strontium quantification in cortical rat bone by time-of-flight secondary ion mass spectrometry. J. R. Soc. Interface.

[60] Rohnke M., Pfitzenreuter S., Mogwitz B., Henss A., Thomas J., Bieberstein D., Gemming T., Otto S.K., Ray S., Schumacher M. (2017). Strontium release from Sr(2+)-loaded bone cements and dispersion in healthy and osteoporotic rat bone. J. Control Release.

[61] Nardone V., Zonefrati R., Mavilia C., Romagnoli C., Ciuffi S., Fabbri S., Palmini G., Galli G., Tanini A., Brandi M.L. (2015). In Vitro Effects of Strontium on Proliferation and Osteoinduction of Human Preadipocytes. Stem Cells Int..

[62] Reitmaier S., Kovtun A., Schuelke J., Kanter B., Lemm M., Hoess A., Heinemann S., Nies B., Ignatius A. (2018). Strontium(II) and mechanical loading additively augment bone formation in calcium phosphate scaffolds. J. Orthop. Res..

[63] Richter R.F., Ahlfeld T., Gelinsky M., Lode A. (2019). Development and Characterization of Composites Consisting of Calcium Phosphate Cements and Mesoporous Bioactive Glass for Extrusion-Based Fabrication. Materials.

[64] Hasan M.L., Kim B., Padalhin A.R., Faruq O., Sultana T., Lee B.T. (2019). In vitro and in vivo evaluation of bioglass microspheres incorporated brushite cement for bone regeneration. Mater. Sci. Eng. C Mater. Biol. Appl..

