# Supplementary material for: Mesoporous Bioactive Glass-Incorporated Injectable Strontium-Containing Calcium Phosphate Cement Enhanced Osteoconductivity in a Critical-Sized Metaphyseal Defect in Osteoporotic Rats
Source: Bioengineering (Basel). 2023 Oct 16;10(10):1203. doi: 10.3390/bioengineering10101203 (PMC10604136; doi:10.3390/bioengineering10101203)
Supplement: Supplementary file 1 [file bioengineering-10-01203-s001.zip › bioengineering-2636953-supplementary.pdf]

Supplemental Data

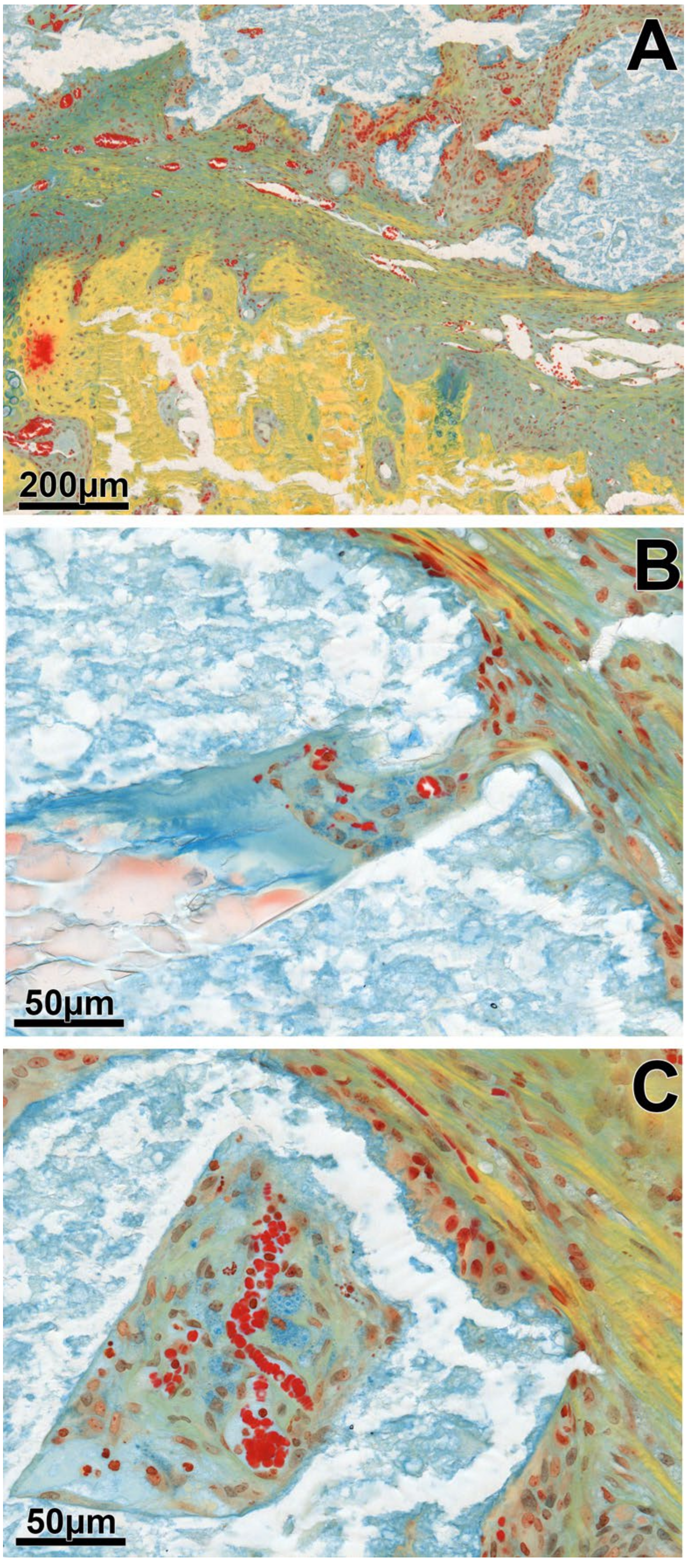

Supplemental Figure S1: *Dissolution of the mesoporous glass allowed the formation of vessels and tissue ingrowth.*

Gene	Forward primer sequence (5‘->3‘)	Reverse primer sequence (5’->3’)	Accession No.	Product length
ALP	atc gga ccc tgc ctt acc	ctc ttg ggc ttg ctg tcg	NM_013059	87
OCN	gag ggc agt aag gtg gtg aa	gtc cgc tag ctc gtc aca at	NM_013414	135
Col10alpha1	cat gtg aag ggg act cac g	gaa gcc tga tcc aag tag cc	XM_002725875	101
Runx2	cca taa cgg tct tca caa atc c	gcg gtc aga gaa caa act agg	NM_053470	137
Col1alpha1	tcc tga cgc atg gcc aag aa	cat agc acg cca tcg cac ac	NM_053304	145
RankL	aaa tta gcg tcc agg tgt cc	ttg aaa gcc cca aag tac g	NM_057149	73
OPG	atg aac aag tgg ctg tgc tg	aaa ggt ttc ctg ggt tgt cc	NM_012870	72
B2M	tgt ctc agt tcc acc cac ct	ggg ctc ctt cag agt gac g	NM_012512.2	191

Supplemental Table S1: *Primer pairs*
